# Postcopulatory Sexual Selection Results in Spermatozoa with More Uniform Head and Flagellum Sizes in Rodents

**DOI:** 10.1371/journal.pone.0108148

**Published:** 2014-09-22

**Authors:** María Varea-Sánchez, Laura Gómez Montoto, Maximiliano Tourmente, Eduardo R. S. Roldan

**Affiliations:** Reproductive Ecology and Biology Group, Museo Nacional de Ciencias Naturales (CSIC), Madrid, Spain; Uppsala University, Sweden

## Abstract

Interspecific comparative studies have shown that, in most taxa, postcopulatory sexual selection (PCSS) in the form of sperm competition drives the evolution of longer and faster swimming sperm. Work on passserine birds has revealed that PCSS also reduces variation in sperm size between males at the intraspecific level. However, the influence of PCSS upon intra-male sperm size diversity is poorly understood, since the few studies carried out to date in birds have yielded contradictory results. In mammals, PCSS increases sperm size but there is little information on the effects of this selective force on variations in sperm size and shape. Here, we test whether sperm competition associates with a reduction in the degree of variation of sperm dimensions in rodents. We found that as sperm competition levels increase males produce sperm that are more similar in both the size of the head and the size of the flagellum. On the other hand, whereas with increasing levels of sperm competition there is less variation in head length in relation to head width (ratio CV head length/CV head width), there is no relation between variation in head and flagellum sizes (ratio CV head length/CV flagellum length). Thus, it appears that, in addition to a selection for longer sperm, sperm competition may select more uniform sperm heads and flagella, which together may enhance swimming velocity. Overall, sperm competition seems to drive sperm components towards an optimum design that may affect sperm performance which, in turn, will be crucial for successful fertilization.

## Introduction

Comparative studies have shown that many taxa respond to increases in sperm competition by increasing sperm size which, in turn, is associated to enhanced sperm swimming speed (mammals: [1]–[4], fish: [5], birds: [6]). Fast swimming velocity is an adaptive trait that maximizes fertilization success in competitive contexts [7]. In mammals, sperm cells increase in total size as a result of an increase in all its components [3]. Longer sperm heads could result in less drag, longer midpieces may contain more mitochondria to generate energy, and an increase in the principal piece would lead to greater propulsive force and, possibly, additional energy production [3], [8]. Further to an elongation of the sperm head, a proportionately longer flagellum (in relation to sperm head length) may better overcome the drag of the sperm head [3], [9], [10]. Studies on closely related species, as well as intraspecific comparisons, have suggested that an early evolutionary step in the overall change of sperm size may be a modification of sperm head dimensions [11], [12] that makes sperm movement more energy-efficient by reducing the degree of resistance offered to the medium in which sperm swim [13]. Modifications in the sperm head may involve changes in elongation [11], an increase in sperm head area, or the development of appendices, such as an apical hook, as seen in rodents [12]. 

Thus, interspecific patterns reveal that sperm competition is a powerful directional selective force driving changes in sperm size. Nevertheless, there are constraints on the maximum size that sperm can achieve, which are imposed by metabolic rate and are therefore body-size dependent [4], [14]. Small-bodied mammals (e.g., rodents and small marsupials), with high mass-specific metabolic rates, are able to respond to increased levels of sperm competition by increasing sperm size, but large mammals cannot.

Much less attention has been paid to intraspecific and intra-male levels of variation in sperm dimensions. In natural populations, males differ in semen quality, sperm dimensions, and sperm swimming velocity, and these differences would have a major impact on male fertility [11], [15]. Theoretical models of evolution of sperm size propose that, under diploid control (i.e., male genotype), a certain sperm size optimum exists for any given level of sperm competition [16]. Such models predict that under intense sperm competition males will be selected to produce sperm of a size close to the optimum, whereas males under less intense selection will not [16]. Thus, greater variation is expected when postcopulatory sexual selection is relaxed than when it is intense.

Support for these predictions comes from a few studies which compared the level of variation between males among insects [17] and passerine birds [18], [19], [20]. These studies found that increased sperm competition reduces variation in sperm size *between* males. However, studies also among passerine birds, which addressed variation *within* males, have generated contradictory results. One study reported a negative relationship between intra-male levels of variation in sperm size and levels of sperm competition [21], whereas others found either weak or non-significant relationships between both variables [19], [20]. Furthermore, considerable sperm variation was found in a highly promiscuous passerine bird [22]. Although it is known that, in mammals, intra-male variation in sperm dimensions is not uncommon [23] relationships between sperm competition levels and sperm variation in size and shape have so far received little attention. In rodents, variation in sperm components has been examined before [24], [25] although no evidence has been provided for a relationship between such variation and sperm competition, with the exception of a recent study which revealed a negative association between sperm competition and variation in hook length [26]. It is worth bearing in mind that mammalian head and flagellum structures are unique [27], [28], so responses to selective pressures could operate differently in mammals when compared to birds.

The aim of this study was to test, in a phylogenetic framework, whether sperm competition reduces variation in the size of spermatozoa produced by individual males in rodents. To this end, we examined a group of muroid rodents that are known to respond to sperm competition by increasing the dimensions of the sperm components [3], [12]. Our study also addressed whether levels of intra-male variation are reduced in traits known to respond fast to increased levels of sperm competition (i.e., dimensions or elongation of the sperm head), in comparison to variation in flagella or proportions between flagellum components.

## Materials and Methods

### Ethics statement

All animal handling was done following Spanish Animal Protection Regulation RD1201/2005, which conforms to European Union Regulation 2003/65. This study was approved by the Ethics Committee of the Spanish Research Council (CSIC). Animals were captured with permissions from Junta de Castilla y León and Comunidad Autónoma de Madrid.

### Sperm preparation and measurements

Sperm dimensions were measured in spermatozoa from adult males of 26 rodent species. *Arvicola terrestris, Arvicola sapidus, Chionomys nivalis, Myodes glareolus, Microtus arvalis, Microtus cabreae, Microtus duodecimcostatus, Microtus lusitanicus *and *Apodemus sylvaticus, *(subfamily Arvicolinae) were trapped in the field during the breeding season (April-June) in different localities of Castilla y León and Comunidad Autónoma de Madrid (Spain) (Table S1). *Lemniscomys barbarus, Mastomys natalensis, Micromys minutus, Mus caroli, Mus macedonicus, Mus minutoides, Mus castaneus, Mus domesticus, Mus musculus, Mus spicilegus, Mus spretus* and *Mus pahari *(subfamily Murinae), as well as *Cricetulus griseus, Mesocricetus auratus, Phodopus campbelli, Phodopus roborovskii *and *Phodopus sungorus *(subfamily Cricetinae) were from wild-derived colonies whose localities of origin are given in Table S1.

Animals were sacrificed by cervical dislocation and immediately weighed. Testes were dissected out and weighed and spermatozoa were obtained and processed as previously described [29]. The following sperm dimensions were quantified: head length (µm), head width (µm), head area (µm^2^), midpiece length (µm), principal plus terminal piece length (µm), total flagellum length (µm) and total sperm length (µm). Head length was measured as the linear distance between the most basal point and the most apical one of the sperm head. Head width was taken as a straight line between the dorsal and ventral regions in the wider region of the sperm head. Head area was measured taking into account the entire sperm head including the apical hook. All measures were taken with the software Image-J v1.41 (NIH, Bethesda, USA).

### Statistical Analyses

Dimensions of all sperm components were obtained for 30 cells from each male (N  =  5 males per species). Coefficient of variation (CV) for each sperm dimension was calculated as follows: CV =  (standard deviation *100)/mean. Coefficient of variation for each male was calculated using sperm dimensions of each individual spermatozoon. Then mean CV for each trait from all males of the same species was calculated. The relation between CVs of six sperm components was also calculated, using the following ratios: CV head length/CV head width, CV midpiece/CV principal piece; CV head length/CV total flagellum length, and CV head area/CV total flagellum length. All variables were transformed using the logarithm of CV to attain normal distributions.

The degree of variation in the sperm head components was compared to that of flagellum ones using paired *t*-tests. Differences in variation patterns in sperm components between the three rodent subfamilies (Arvicolinae, Cricetinae and Murinae) were analyzed using Analysis of Variance with phylogenetic correction (PGLS ANOVA). 

To test whether different levels of sperm competition were associated with intra-male variation in sperm dimensions across species, multiple regression analysis were performed using the CV of the different sperm components and ratios as dependent variables. Body mass and testes mass were used as predictor variables. The increases in testes size in relation to body size is an almost universal response to increased levels of sperm competition in polyandrous species [30], [31]. Thus, testes mass corrected for body mass (hereafter, relative testes mass) is a reliableproxy of sperm competition levels which has been used before in comparative studies in Muroid rodents [3], [12].

**Figure 1:**
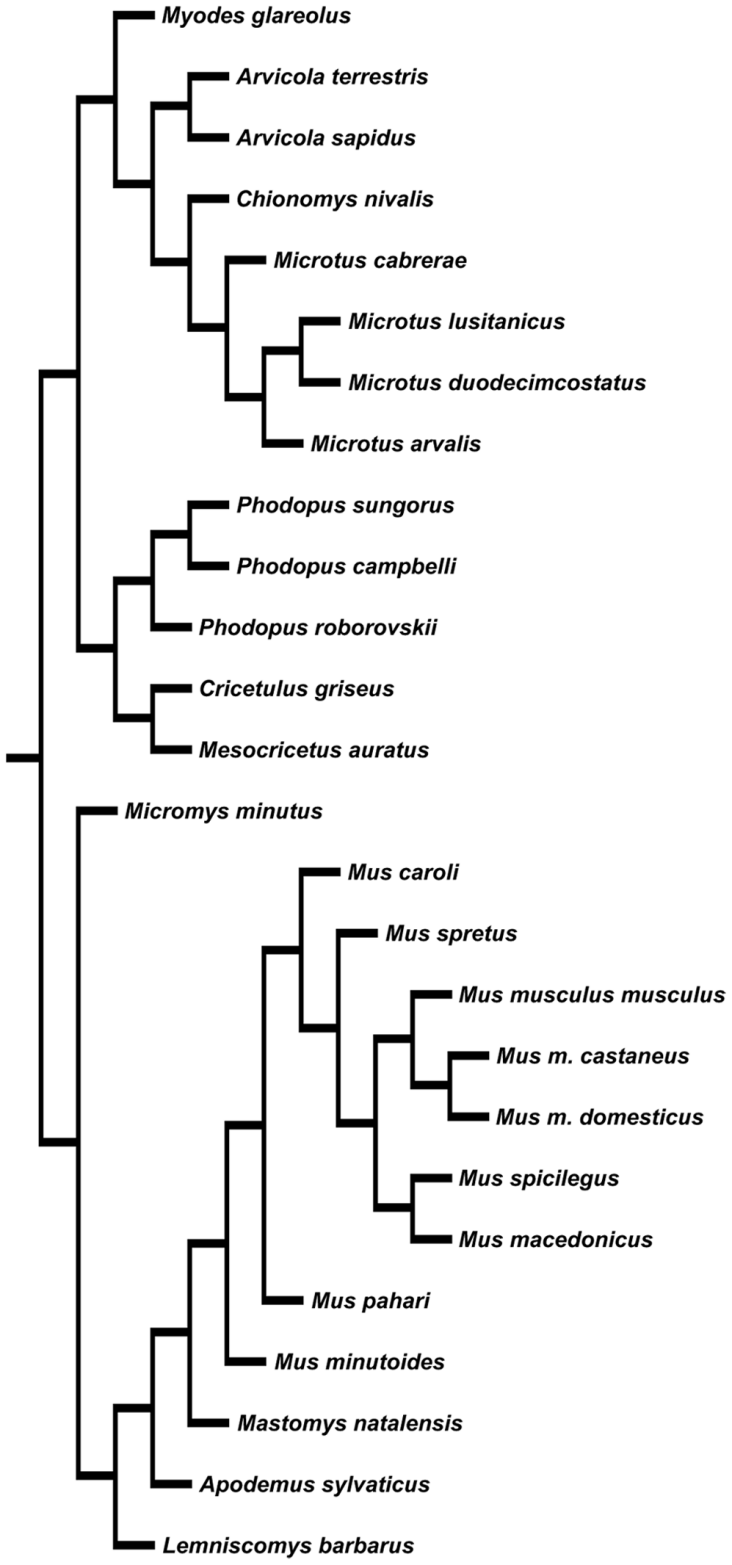
Phylogeny of 26 rodent species used in this study. The phylogenetic tree has been reconstructed from published data [32].

Because species trait values may be similar as a result of phylogenetic association rather than selective evolution, regression analyses were performed using a generalized least-squares approach in a phylogenetic framework (PGLS). PGLS incorporates phylogenetic interdependency among the data points by including the phylogenetic structure within a standard linear model. Subsequently, PGLS estimates a phylogenetic scaling parameter lambda (λ) of the tree's branch lengths that fits evolution proceeding via Brownian motion. If λ values are close to 0, the variables are likely to have evolved independently of phylogeny, whereas λ values close to 1 indicate strong phylogenetic association of the variables. The phylogenetic reconstruction for species analysed in this study (Fig. 1) was inferred from a phylogenetic hypothesis which was based on 11 nuclear and mitochondrial genes [32]. Statistical analyses were conducted with Caper v 0.5 (Orme et al. 2012) (R Foundation for Statistical Computing) and with InfoStat v.2013p (FCA, Universidad Nacional de Cordoba, Argentina). *P-*values were considered statistically significant at α<0.05. Data were plotted as partial residual graphics, using the residual testis mass as an indicator of sperm competition level.

## Results

Testes mass relative to body mass was positively associated to total sperm length among species included in this study (*F* = 16.87, *P* = 0.0004, d.f. = 1), in agreement with our earlier analyses [3], [14]. Data of intra-male coefficient of variation of the different sperm components in 26 rodent species are shown in Table S2. Paired *t*-tests revealed that sperm head dimensions (head length, head width and head area) had a higher degree of variation than that observed for dimensions of flagellum components. This was observed for the three lineages: murids (*t* = 10.71, *P*<0.001, d.f. = 25), arvicolids (*t* = 4.76, *P* = 0.002, d.f. = 25) and cricetids (*t* = 4.71, *P* = 0.009, d.f. = 25). Among the three rodent subfamilies analyzed, Arvicolinae showed the highest CV values, which were statistically different for CVs of sperm head length and sperm area from the other two subfamilies (Cricetinae and Murinae). No statistically significant differences were found between the variation in flagellum components of the three subfamilies, with the exception of the CV of the midpiece length which was significantly higher in Arvicolinae than in Murinae (Table 1).

**Table 1:** Intra-male coefficient of variation for three rodent subfamilies.

Subfamily	CV head length	CV head width	CV head area	CV midpiece length	CV principal piece length	CV total flagellum length	CV total sperm length
Arvicolinae (N = 13)	6.45 (±0.151)^*,*^	8.00 (±0.241)^†,*^	10.00 (±0.300)^*,*^	7.97 (±0.303)^†,*^	4.12 (±0.132)^†,†^	2.96 (±0.114)^†,†^	2.78 (±0.107)^†,†^
Cricetinae (N = 5)	3.81 (±0.291)	7.19 (±0.987)	5.90 (±0.336)	4.69 (±0.686)	2.41 (±0.363)	1.19 (±0.137)	1.14 (±0.127)
Murinae (N = 8)	3.81 (±1.321)	5.05 (±1.260)	5.69 (±1.879)	3.51 (±1.477)	2.30 (±0.867)	1.79 (±0.836)	1.68 (±0.786)
All rodents	4.62 (±0.467)	6.37 (±0.504)	7.06 (±0.695)	5.11 (±0.614)	2.88 (±0.317)	2.04 (±0.285)	1.92 (±0.267)

Data are average (± S.E.M.) The superscripts in Arvicolidae parameters indicate whether there are differences (^†^ not significant; *P<0.05; PGLS ANOVA) when compared with Cricetinae (first position) or Murinae (second position).

**Figure 2:**
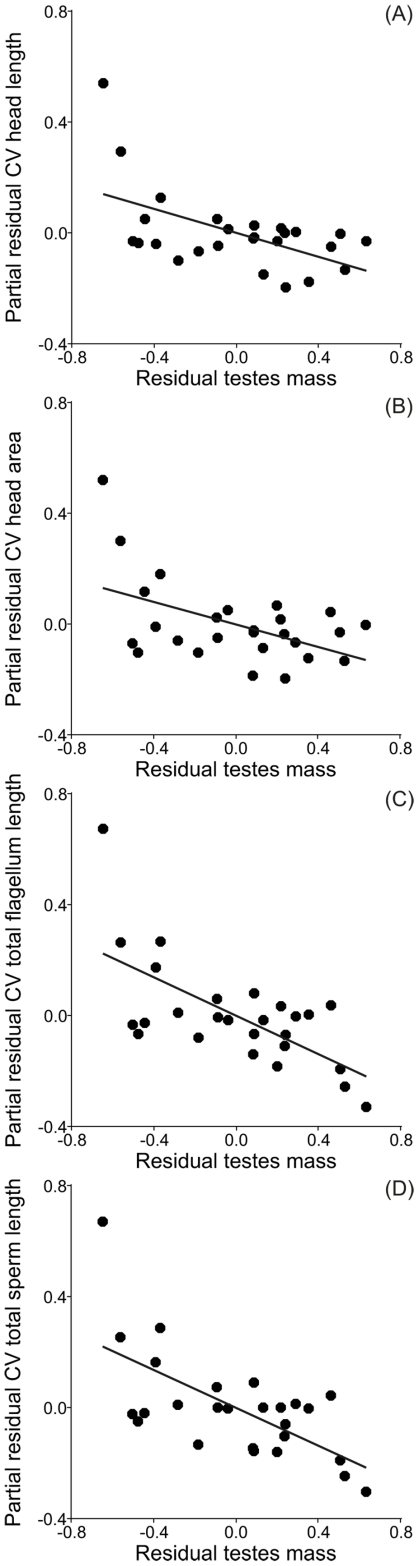
Relationships between residual testes mass and coefficients of variation (CV) for dimensions of several sperm components. (A) CV head length, (B) CV head area, (C) CV total flagellum length, and (D) CV total sperm length.

Multiple regression analysis, controlling for phylogenetic effects, revealed a significant and negative relationship between testes mass corrected for body mass and intra-male CV of head length, head area, total flagellum length and total sperm length (Table 2, Fig. 2). Head width showed a similar trend, but results did not reach statistical significance (Table 2).

**Table 2:** Phylogenetically-controlled multiple regression analyses of coefficient of variation (CV) of sperm dimensions in relation to body mass and testes mass.

Dependent variable	Predictor	Adjusted *R* ^2^	Slope	*F*	*P*	Lambda (λ)	Effect size (r)	Effect size CLs
**CV HL**	Body mass	0.33	0.20	0.007	0.93	0.47^*,*^	0.01	(−0.39 to 0.42)
	Testes mass		−0.23	14.69	**<0.001**		0.62	**(0.32 to 1.14)**
**CV HW**	Body mass	0.06	0.13	0.02	0.88	0.79^*,†^	0.03	(−0.37 to 0.43)
	Testes mass		−0.14	3.69	0.06		0.37	(−0.01 to 0.79)
**CV HA**	Body mass	0.20	0.24	0.72	0.40	0.38^†,*^	0.17	(−0.23 to 0.58)
	Testes mass		−0.20	7.77	0.01		0.50	**(0.14 to 0.96)**
**CV MPL**	Body mass	−0.07	0.06	0.003	0.95	0.68^*,†^	0.01	(−0.39 to 0.41)
	Testes mass		−0.06	0.27	0.60		0.10	(−0.30 to 0.51)
**CV PPL**	Body mass	0.05	0.03	0.97	0.33	0.95^*,†^	0.20	(−0.20 to 0.61)
	Testes mass		−0.13	2.44	0.13		0.31	(−0.08 to 0.72)
**CV TFL**	Body mass	0.37	0.17	2.42	0.13	0.17^†,*^	0.30	(−0.09 to 0.72)
	Testes mass		−0.31	14.82	**<0.001**		0.62	(0.32 to 1.14)
**CV TSL**	Body mass	0.36	0.19	1.86	0.18	0.16^†,*^	0.27	(−0.12 to 0.68)
	Testes mass		−0.31	14.53	**<0.001**		0.62	(0.31 to 1.13)

Data have been processed as the logarithm of CV. All tests were conducted with 23 df. The superscripts following λ value indicate significance levels (^†^ n.s.; *P<0.05) in a likelihood ratio tests against models with λ = 0 (first position) and λ = 1 (second position). The effect size *r* was calculated from the *F* values; its noncentral 95% confidence limits (CLs) are also given. Confidence intervals excluding 0 indicate statistically significant relationships. *P* values and CLs that indicate statistical significance are shown in bold. Abbreviations: HL, head length; HW, head width; HA, head area; MPL, midpiece length; PPL, principal piece length; TFL, total flagellum length; TSL, total sperm length.

**Figure 3:**
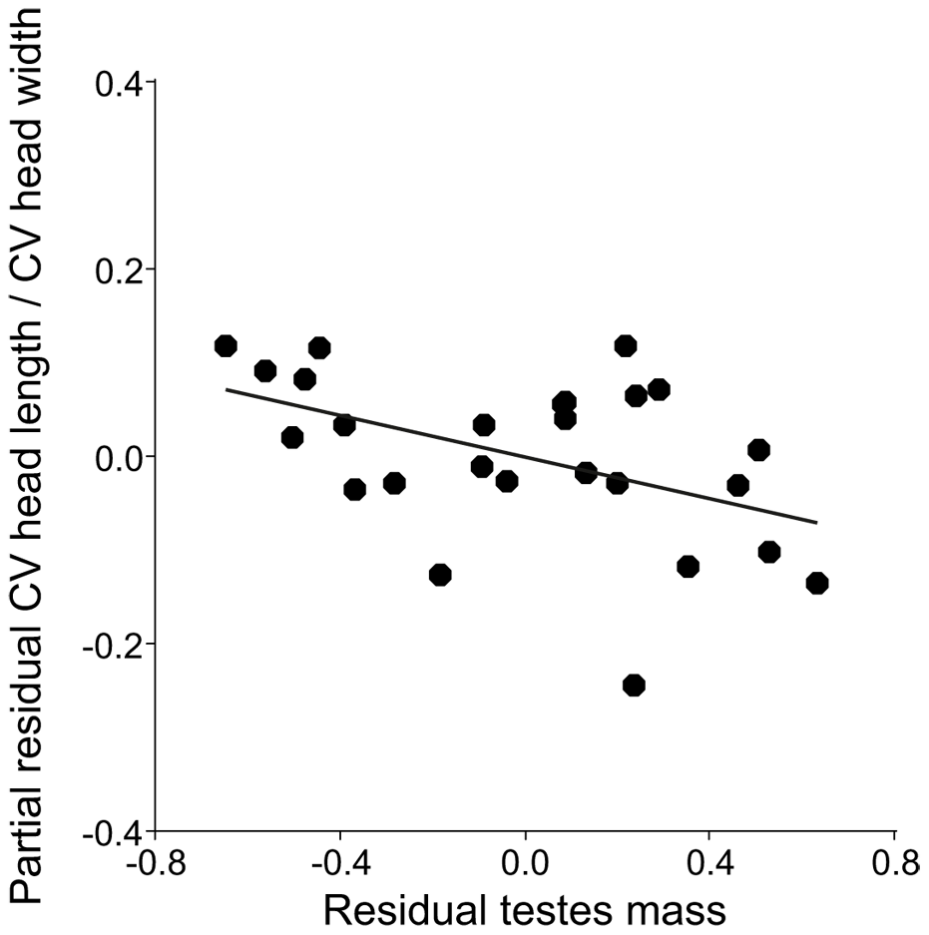
Relationship between residual testes mass and the ratio between coefficients of variations (CV) of head length and head width of several sperm components.

Significant negative relationships were found in the regression analyses between testes mass corrected for body mass and the intra-male ratio of CV head length/CV head width (Table 3, Fig. 3). There were no relations with intra-male CV for the ratios CV midpiece length/CV principal piece length, CV head length/CV total flagellum length or CV head area/CV total flagellum length (Table 3).

**Table 3:** Phylogenetically-controlled multiple regression analyses of coefficient of variation (CV) of ratios between dimensions of sperm components in relation to body mass and testes mass.

Dependent variable	Predictor	Adjusted *R* ^2^	Slope	*F*	*P*	Lambda (λ)	Effect size(r)	Effect size CLs
CV HL/CV HW	Body mass	0.32	0.12	0.76	0.39	0.00^†,*^	0.17	(−0.39 to 0.42)
	Testes mass		−0.16	13.31	**0.001**		0.60	**(0.29 to 1.10)**
CV HL/CV TFL	Body mass	0.17	0.03	4.77	**0.03**	0.00^†,*^	0.41	**(0.03 to 0.84)**
	Testes mass		0.10	2.68	0.11		0.32	(−0.07 to 0.73)
CV HA/CV TFL	Body mass	0.33	0.08	10.86	**0.003**	0.00^†,*^	0.56	**(0.23 to 1.05)**
	Testes mass		0.12	3.50	0.07		0.36	(−0.02 to 0.78)
CV MPL/CV PPL	Body mass	0.07	0.11	3.74	0.06	0.00^†,*^	0.37	(−0.01 to 0.80)
	Testes mass		0.02	0.12	0.72		0.07	(−0.33 to 0.48)

Data have been processed as the logarithm of the CV of ratios between sperm components. All tests were conducted with 23 df. The superscripts following λ value indicate significance levels (^†^ n.s.; *P<0.05) in a likelihood ratio tests against models with λ = 0 (first position) and λ = 1 (second position). The effect size *r* was calculated from the *F* values; its noncentral 95% confidence limits (CLs) are also given. Confidence intervals excluding 0 indicate statistically significant relationships. *P* values and CLs that indicate statistical significance are shown in bold. Abbreviations: HL, head length; HW, head width; HA, head area; MPL, midpiece length; PPL, principal piece length; TFL, total flagellum length.

## Discussion

The results of this study show that as sperm competition increases rodent males produce sperm that are more uniform in size. This reduction in the degree of variation was observed both for the dimensions of sperm flagellum and sperm head and, hence, in total length of spermatozoa. In general terms, variation in dimensions was higher for the sperm head than for the sperm flagellum. Increases in sperm competition were also associated with lower variation in the ratio between the CVs of head length and head width (CV head length/CV head width).

Our findings suggest that the same selective force that drives differences between species in total sperm length may also be responsible for a reduced variation in sperm dimensions within males, i.e. directional sexual selection. The fact that sperm components become more similar as they increase in size, suggests that sperm competition acts by tightening controls during sperm production. Two studies in rodents at the intraspecific level [24], [25] provided evidence for this idea. Their findings revealed that variation in size and shape of sperm components is higher with low levels of sperm competition and hence, in absence of sperm competition the investment on high quality sperm production is not advantageous, so this variation may be due to a relaxation in the control of spermatogenesis.

Modifications in sperm head size and shape may be achieved by rapid evolutionary changes in genes controlling chromatin condensation and nucleus size, such as the protamine genes which, as sperm competition increases, may become more efficient at condensing DNA, resulting in more compact nuclei and more streamlined heads [33]. Less divergent sequences between protamine 1 and cleaved protamine 2 were found associate with smaller and more elongated heads relative to the total sperm length [34]. Altogether, these results suggest that once the optimum design to perform in a sperm competition context has been achieved, proteins involved in shaping the sperm head tend to reduce the degree of variation in their sequences. On the other hand, the increase in length of the flagellum until an optimum size is reached, as revealed by a smaller CV of this sperm component, may provide more efficient thrust to propel the cell, increasing swimming speed [1], [5], [6] and improving the chances of fertilization success [7].

No relationship was found between levels of sperm competition and the ratios CV midpiece/CV principal piece, or CV head length/CV total flagellum length. On the other hand, as sperm competition increased, the degree of variation in head length in relation to head width (CV head length/CV head width) decreased significantly. These results suggest that variation in sperm components may exhibit different responses to sperm competition. Overall, head length seems to be the variable that is more sensitive to postcopulatory sexual selection, perhaps due to a major relevance for swimming speed [11].

In comparative analyses of bird species, increases in total sperm length are accompanied by decreases in the degree of variation in total sperm size, and this has been interpreted to result from postcopulatory sexual selection [19], [21]. A similar association between increases in sperm length and decreases in variation has also been observed between populations of the same bird species [35], suggesting that a tightening in the control of the spermatogenic process may first target events at a specific level. As a result, sperm components may not respond simultaneously to increases in sperm competition. Enlargement of the sperm head or flagellum could occur rather independently and, similarly, a higher uniformity in dimensions of each of these components may not necessarily occur concomitantly. A study of evolutionary trajectories of different sperm traits in birds revealed that the midpiece and the flagellum respond to selection in a similar way, whereas head length differs in its response [36]. Our results from rodents, with a higher degree of variation among sperm head measures compared to flagellum dimensions, agree with the findings in birds, supporting the hypothesis of an independent evolution of sperm components [36]. In an evolutionary framework, sperm competition seems to play a stabilizing role, leading sperm cells to increase in overall size towards an optimum. This optimum is likely to be a balance between the level of sperm competition and the energetic budget determined by the metabolic rate [4], [14].

The opposite pattern is observed when the control of spermatogenesis is negatively affected by genetic stress. This is the case among inbred males which show an increase in the proportion of morphologically abnormal sperm and therefore an increase in the diversity of sperm sizes and shapes within males [37]. This may also be true when levels of sperm competition are relaxed and sperm become pleiomorphic (or abnormal) and the diversity in sperm shape and size increases. Sperm phenotypic plasticity under low sperm competition levels has been documented in species from different rodent genera [23], [38], [39]. On this study, we observed sperm pleiomorphism in *Microtus duodecimcostatus*, a species having low sperm competition levels and sperm with high CVs in dimensions of all sperm components. These observations point to sperm competition as a stabilizing selection pressure in the degree of sperm size and shape variation.

Sperm morphology is thought to be a key determinant of sperm performance in relation to fertilization success. However, recent findings have shown that differences in sperm function within an ejaculate, such as sperm longevity, may play a crucial role in offspring development [40]. This result is important for two reasons. First, the reduction in the degree of variation might be a universal phenomenon for all sperm features. Second, sperm competitiveness may be relevant for fertilization success, but it may also influence post-fertilization processes.

We conclude that as sperm competition increases, sperm components not only respond by increasing in size but they also show a reduced degree of variation within males. In rodents, the response to postcopulatory sexual selection is a modification in sperm flagellum dimensions so that all spermatozoa get close to an optimal length, presumably because it results in more efficient use of energy resources that provides more thrust and enhances swimming speed. This may be accompanied by an enlargement of the sperm head and a reduction in its variation, so that all spermatozoa share a similar design to achieve more efficient swimming. These findings have major implications for our understanding of how postcopulatory sexual selection operates upon the size of different sperm components and the control of spermatogenesis.

## Supporting Information

Table S1 **Localities of origin of muroid rodents used in this study.**
(DOCX)

Table S2 **Intra-male coefficients of variation (CV) of sperm components in muroid rodents examined in this study.**
(DOCX)
