# Supplementary material for: Postcopulatory Sexual Selection Results in Spermatozoa with More Uniform Head and Flagellum Sizes in Rodents
Source: PLoS One. 2014 Sep 22;9(9):e108148. doi: 10.1371/journal.pone.0108148 (PMC4171531; doi:10.1371/journal.pone.0108148)
Supplement: Table S1 — Localities of origin of muroid rodents used in this study. (DOCX) [file pone.0108148.s001.docx]

**Table S1.** Localities of origin of muroid rodents used in this study.

| **Species** | **Capture location** |
| --- | --- |
| *Apodemus sylvaticus* | Cerrato, Castilla-León, Spain |
| *Arvicola sapidus* | Soria, Castilla-León, Spain |
| *Arvicola terrestris* | Palencia, Castilla-León, Spain |
| *Chionomys nivalis* | Navacerrada, Madrid, Spain |
| *Cricetulus griseus* | Kumtagh, China |
| *Lemniscomys barbarus* | N. of Atlas Mountains, Morocco |
| *Mastomys natalensis* | Durban, South Africa |
| *Mesocricetus auratus* | Aleppo, Syria |
| *Micromys minutus* | Ulyanovsk, Russia |
| *Microtus arvalis* | Castilla-León, Spain |
| *Microtus cabrerae* | Chapinería, Madrid, Spain |
| *Microtus duodecimcostatus* | Castilla-León, Spain |
| *Microtus lusitanicus* | El Ventorrillo, Madrid, Spain |
| *Mus bactrianus* | Machad, Iran |
| *Mus caroli* | Khorat, Thailand |
| *Mus castaneus* | Masinagudi, India |
| *Mus cookii* | Thailand |
| *Mus domesticus* | Azzemour, Morocco |
| *Mus famulus* | Kotagiri, India |
| *Mus macedonicus* | Slantchev Briag, Bulgary |
| *Mus minutoides* | Cape Town, South Africa |
| *Mus musculus* | Alazani, Georgia |
| *Mus pahari* | Tak, Thailand |
| *Mus spicilegus* | Kalomeyevka, Ukraine |
| *Mus spretus* | Azzemour, Morocco |
| *Myodes glareolus* | Montseny, Barcelona, Spain |
| *Phodopus campbelli* | Altai, Mongolia |
| *Phodopus roborovskii* | Tuva, Russia |
| *Phodopus sungorus* | Baraba Steppe, Kazakhstan |
