# Supplementary material for: Postcopulatory Sexual Selection Results in Spermatozoa with More Uniform Head and Flagellum Sizes in Rodents
Source: PLoS One. 2014 Sep 22;9(9):e108148. doi: 10.1371/journal.pone.0108148 (PMC4171531; doi:10.1371/journal.pone.0108148)
Supplement: Table S2 — Intra-male coefficients of variation (CV) of sperm components in muroid rodents examined in this study. (DOCX) [file pone.0108148.s002.docx]

| **Species** | **CV head length** | **CV head width** | **CV head area** | **CV midpiece**  **length** | **CV principal piece** | **CV total flagellum**  **length** | **CV total sperm length** |
| --- | --- | --- | --- | --- | --- | --- | --- |
| *Apodemus sylvaticus* | 3.84 | 5.77 | 7.19 | 3.05 | 2.41 | 1.95 | 1.88 |
| *Arvicola sapidus* | 4.32 | 5.77 | 7.02 | 5.65 | 2.13 | 1.51 | 1.50 |
| *Arvicola terrestris* | 5.11 | 5.55 | 9.50 | 2.27 | 1.85 | 1.55 | 1.52 |
| *Chionomys nivalis* | 4.58 | 4.93 | 7.12 | 6.76 | 2.92 | 1.87 | 1.64 |
| *Cricetulus griseus* | 4.04 | 7.73 | 6.55 | 4.28 | 3.32 | 0.84 | 0.83 |
| *Lemnyscomys barbarus* | 4.20 | 5.21 | 4.40 | 3.06 | 1.78 | 1.26 | 1.18 |
| *Mastomys natalensis* | 4.69 | 7.07 | 8.03 | 3.26 | 2.32 | 1.61 | 1.59 |
| *Mesocricetus auratus* | 3.09 | 5.81 | 5.64 | 6.56 | 3.12 | 1.62 | 1.54 |
| *Micromys minutus* | 4.24 | 5.29 | 5.35 | 3.27 | 3.03 | 2.40 | 2.26 |
| *Microtus arvalis* | 4.92 | 7.10 | 7.11 | 9.48 | 3.62 | 2.01 | 1.96 |
| *Microtus cabrerae* | 8.64 | 9.84 | 13.48 | 8.33 | 4.81 | 3.19 | 2.97 |
| *Microtus duodecimcostatus* | 14.91 | 15.89 | 21.54 | 16.81 | 9.37 | 8.50 | 7.95 |
| *Microtus lusitanicus* | 5.56 | 8.35 | 9.40 | 7.89 | 5.39 | 3.48 | 3.39 |
| *Myodes glareolus* | 3.56 | 6.60 | 4.88 | 6.52 | 2.86 | 1.56 | 1.29 |
| *Mus caroli* | 4.04 | 4.90 | 5.95 | 4.32 | 1.97 | 1.60 | 1.21 |
| *Mus musculus castaneus* | 3.64 | 5.28 | 6.43 | 6.29 | 1.99 | 1.35 | 1.30 |
| *Mus musculus domesticus* | 3.85 | 4.42 | 4.89 | 3.55 | 1.91 | 1.61 | 1.55 |
| *Mus musculus musculus* | 3.85 | 4.96 | 6.20 | 4.85 | 3.44 | 2.76 | 2.52 |
| *Mus macedonicus* | 3.34 | 4.96 | 5.50 | 2.91 | 2.38 | 1.89 | 1.77 |
| *Mus minutoides* | 4.01 | 5.36 | 5.58 | 3.13 | 2.26 | 1.66 | 1.60 |
| *Mus pahari* | 4.21 | 4.93 | 5.30 | 2.31 | 2.25 | 1.87 | 1.80 |
| *Mus spicilegus* | 2.94 | 4.24 | 5.07 | 2.26 | 2.26 | 1.81 | 1.75 |
| *Mus spretus* | 2.73 | 3.28 | 4.13 | 3.35 | 1.82 | 1.54 | 1.48 |
| *Phodopus campbelli* | 4.37 | 6.06 | 6.37 | 2.38 | 1.55 | 1.11 | 1.06 |
| *Phodopus roborovskii* | 3.13 | 5.51 | 4.71 | 5.21 | 1.66 | 1.01 | 0.97 |
| *Phodopus sungorus* | 4.39 | 10.83 | 6.24 | 5.01 | 2.39 | 1.35 | 1.30 |

**~~Table 1.~~** ~~Intra-male coefficients of variation (CV) of seven sperm components in 26 rodent species.~~

**Table S2.** Intra-male coefficients of variation (CV) of sperm components in muroid rodents examined in this study.
